# Commentary: Pingwei Powder alleviates high-fat diet-induced colonic inflammation by modulating microbial metabolites SCFAs

**DOI:** 10.3389/fcimb.2025.1709791

**Published:** 2025-10-20

**Authors:** Kangxiao Guo, Xuehuan He, Junping Zou, Yuan Tang

**Affiliations:** ^1^ Changsha Health Vocational College, Changsha, China; ^2^ Changjun Bilingual Xingsha School, Changsha, China

**Keywords:** Pingwei Powder, high-fat diet, colonic inflammation, gut microbiota, short-chain fatty acids, PI3K/AKT/mTOR pathway, autophagy

## 1 Introduction

Ulcerative colitis (UC), a major subtype of inflammatory bowel disease (IBD), is characterized by chronic relapsing inflammation of the colonic mucosa, presenting with symptoms such as persistent diarrhea, hematochezia, and abdominal pain (Wangchuk et al., 2024). By 2023, the global prevalence of UC had exceeded 5 million cases, imposing a substantial burden on healthcare systems and patient quality of life (Wangchuk et al., 2024). While the pathogenesis of UC remains incompletely understood, accumulating evidence highlights the critical role of environmental factors, especially particularly dietary patterns, in disease initiation and progression. High-fat diets (HFDs), defined as diets containing>30% energy from fat, have emerged as a key risk factor: epidemiological studies involving 170, 805 participants demonstrated that high intake of trans-unsaturated fatty acids increases the risk of UC by 40% (Ananthakrishnan et al., 2014). Mechanistically, HFDs disrupt gut homeostasis by inducing gut microbiota dysbiosis, impairing intestinal barrier function, and triggering low-grade systemic inflammation (Fritsch et al., 2021; Hyun, 2021).

Current therapeutic options for UC are limited by efficacy gaps and high costs: conventional medications such as 5-aminosalicylic acid (5-ASA) only induce remission in 40–50% of patients, while biological agents are prohibitively expensive for most populations (Gao et al., 2019). In contrast, traditional Chinese medicine (TCM) formulas have gained attention for their cost-effectiveness and multi-target therapeutic effects in UC management. Pingwei Powder (PWP), a classic TCM formula first documented in the Taiping Huimin Heji Jufang, which is Formulas from the Imperial Pharmacy for Universal Relief, of the Song Dynasty, consists of four herbs, Cang Zhu, Hou Po, Chen Pi and Gan Cao. For over a millennium, PWP has been used to treat digestive disorders associated with “dampness retention” in TCM, and modern studies have confirmed its anti-inflammatory, intestinal barrier-protective, and gut microbiota-regulating properties (Fan et al., 2023; Zhang et al., 2023, 2019).

A recent landmark study by Liu et al. (2025) published in *Frontiers in Cellular and Infection Microbiology* systematically explored the therapeutic mechanism of PWP in HFD-induced colonic inflammation. Using a murine model of UC induced by HFD combined with dextran sulfate sodium (DSS), the study demonstrated that PWP alleviates colonic inflammation by enhancing the abundance of short-chain fatty acid (SCFA)-producing gut bacteria, increasing intestinal SCFA levels, inhibiting the PI3K/AKT/mTOR pathway, and promoting colonic epithelial autophagy. This finding aligns with a growing body of evidence highlighting the pivotal role of SCFAs in TCM formula-mediated gut homeostasis regulation. For instance, Guo et al. (2025) recently reported that sodium propionate synergizes with Sishen Pill to regulate gut microbiota in diarrhea with kidney-yang deficiency syndrome, reducing the required dosage of the TCM formula while maintaining efficacy. Such studies collectively underscore the potential of SCFA-based synergistic strategies to optimize TCM therapeutic effects.

Notably, compared with other TCM formulas for UC, PWP exhibits unique advantages in targeting HFD-related pathogenesis. For example, Huangqin Decoction primarily exerts therapeutic effects by regulating amino acid metabolism and inhibiting the mTOR pathway (Li et al., 2022), but it lacks a direct regulatory effect on SCFA-producing bacteria— a core mechanism of PWP in counteracting HFD-induced gut dysbiosis. Sishen Pill, while requiring SCFA synergy to enhance efficacy (Guo et al., 2025), focuses on “kidney-yang deficiency” syndrome and is less targeted at the “dampness retention” associated with HFD-induced metabolic disturbances, which PWP is traditionally indicated for. This comparison highlights PWP’s distinct value in treating HFD-related UC by integrating SCFA modulation and syndrome-specific efficacy.

This commentary synthesizes the key findings of Liu et al.’s study, evaluates its strengths and limitations, and proposes future research directions to advance the clinical translation of PWP in UC treatment.

## 2 General comments

### 2.1 Strengths of Liu et al.’s study

#### 2.1.1 Innovative integration of network pharmacology and experimental validation

Liu et al. (2025) adopted a “target prediction-experimental verification” approach that aligns with modern TCM research paradigms. First, using the TCM Systems Pharmacology Database (TCMSP), the authors identified 104 active components of PWP (with oral bioavailability ≥30% and drug-likeness ≥0.18) and 235 unique pharmacological targets. By intersecting these targets with 5,510 UC-associated targets from databases including OMIM and Gene Cards, they identified 177 potential therapeutic targets for PWP. KEGG pathway enrichment analysis of these targets revealed significant enrichment in the PI3K/AKT/mTOR pathway—a key regulator of UC pathogenesis.

Subsequent animal experiments validated this prediction: PWP significantly downregulated the expression of PI3K protein and the phosphorylation ratios of AKT and mTOR in colonic tissues of UC mice. This integrative approach not only provides a scientific basis for PWP’s “multi-component, multi-target” effects but also avoids the limitations of traditional experimental research. Similar network pharmacology-based strategies have been successfully applied to other TCM formulas for UC, such as Huangqin Decoction, confirming its reliability (Li et al., 2022).

#### 2.1.2 Comprehensive evaluation of gut microbiota-metabolite-pathway axis

A major contribution of Liu et al.’s study is its systematic exploration of the “gut microbiota-SCFAs-PI3K/AKT/mTOR-autophagy” regulatory axis, a core mechanism of PWP’s therapeutic effect. The authors employed a multimodal approach to characterize this axis. 16S rDNA sequencing showed that PWP reversed HFD-DSS-induced gut dysbiosis by reducing the abundance of opportunistic pathogens and increasing the levels of SCFA-producing bacteria. Linear discriminant effect size (LEfSe) analysis further confirmed these taxonomic shifts, highlighting PWP’s selective modulation of gut microbiota. Gas chromatography-mass spectrometry (GC/MS) revealed that PWP significantly increased intestinal levels of butyric acid, valeric acid, and isovaleric acid which is the key SCFAs with anti-inflammatory and barrier-protective effects. Correlation analysis showed that the abundance of Alistipesand Parabacteroides was positively correlated with SCFA levels and negatively correlated with colonic pro-inflammatory cytokine expression, establishing a link between microbiota and metabolite changes. Notably, this SCFA-mediated microbiota regulation is not unique to PWP, Guo et al. (2025) similarly found that sodium propionate enhances the gut microbiota-regulating effect of Sishen Pill, increasing the abundance of beneficial bacteria such as *Lactobacillus johnsonii* and *Bifidobacterium*, this finding that supports the universal role of SCFAs in TCM formula-mediated gut health maintenance. Western blotting demonstrated that PWP inhibited PI3K/AKT/mTOR activation, which in turn upregulated autophagy markers and downregulated the autophagy substrate P62. Transmission electron microscopy (TEM) further visualized the increase in autophagosome number in PWP-treated colonic epithelial cells, confirming enhanced autophagic activity. This comprehensive analysis addresses a critical gap in TCM research—many previous studies only reported phenotypic changes without elucidating the underlying molecular and microbial mechanisms (Qu et al., 2022).

#### 2.1.3 Rigorous validation using fecal microbiota transplantation and SCFA supplementation

To confirm the causal role of gut microbiota and SCFAs in PWP’s efficacy, Liu et al. (2025) performed two key validation experiments. One is Fecal microbiota transplantation (FMT). Mice receiving fecal enemas from PWP-treated mice (FMT-PWP group) exhibited significantly attenuated colonic inflammation, higher expression of tight junction proteins, and inhibited PI3K/AKT/mTOR activation compared to mice receiving feces from model mice (FMT-Mol group). This directly demonstrated that PWP-modulated gut microbiota is sufficient to mediate anti-inflammatory effects. Another is butyrate supplementation test. In pseudo-germ-free mice (treated with antibiotics), supplementation with sodium butyrate (ABX-PWP-BA group) enhanced the therapeutic effect of PWP, compared to the ABX-PWP group, the ABX-PWP-BA group showed reduced colonic injury, upregulated SCFA receptor GPR43 expression (Kimura et al., 2013), and further enhanced autophagy. These results confirm that butyrate, the most biologically active SCFA, is a key mediator of PWP’s efficacy. This finding is consistent with Guo et al.’s (2025) observation that sodium propionate acts as a booster for TCM formulas, enhancing their therapeutic effects by modulating gut microbiota and metabolite levels.

Such validation experiments are rare in TCM studies but are essential for establishing causality rather than correlation. Similar approaches have been used to confirm the role of microbiota in other herbal interventions, such as the effect of Bacteroides fragilis on renal fibrosis (Zhou et al., 2022), highlighting their scientific rigor.

4. Detailed Characterization of PWP’s Chemical Composition

The authors conducted ultra-performance liquid chromatography-tandem mass spectrometry (UPLC/MS/MS) analysis to identify 15 key components of PWP, including atractyloside A, neohesperidin, liquiritigenin, and honokiol. This chemical characterization is critical for two reasons: first, it ensures the quality and reproducibility of PWP used in the study. Second, it provides a basis for future studies on the structure-activity relationships of PWP’s components. For example, hesperidin has been shown to reduce DSS-induced murine colitis by inhibiting oxidative stress (Xu et al., 2009), while glycyrrhizic acid (a triterpenoid in Glycyrrhiza uralensis) mitigates inflammation by regulating NF-κB signaling (Khan et al., 2013). Liu et al.’s identification of these components lays the groundwork for dissecting the individual and synergistic effects of PWP’s herbs.

However, it is important to note that TCM quality control was not fully addressed in this study. PWP’s efficacy is highly dependent on the quality of its constituent herbs, which can vary significantly due to factors such as geographical origin, harvesting time, and processing methods. For instance, Hou Po exhibits substantial batch-to-batch variation in the content of its active components magnolol and honokiol (Luo et al., 2019), which are key anti-inflammatory constituents of PWP. Similarly, Cang Zhu (Atractylodes lancea) from different regions shows differences in atractyloside A levels, a component linked to PWP’s gut microbiota-regulating effects (Zhang et al., 2023). Without standardized quality control protocols, the reproducibility of PWP’s clinical efficacy may be compromised. This gap weakens the practical guidance for PWP’s clinical application and warrants attention in future research.

### 2.2 Limitations of Liu et al.’s study

Despite its significant contributions, Liu et al.’s study has several limitations that warrant consideration:

#### 2.2.1 Incomplete analysis of gut microbiota function

While 16S rDNA sequencing provided insights into microbial community structure, the study did not employ metagenomic sequencing—a technique that reveals the functional potential of the gut microbiome. For example, metagenomic sequencing could identify changes in genes encoding butyrate-producing enzymes or LPS biosynthesis genes in pathogenic bacteria (Ma et al., 2024). A recent study by Ma et al. (2024) demonstrated that metagenomic analysis of gut microbiota from pregnant women uncovered functional pathways not detected by 16S rRNA sequencing. For PWP research, metagenomics would clarify whether PWP modulates SCFA levels by altering microbial functional genes or simply by changing taxonomic abundance.

Additionally, the study focused on bacterial communities but ignored other gut microbes such as fungi and archaea. Emerging evidence suggests that fungal dysbiosis contributes to UC pathogenesis by triggering immune responses (Wang et al., 2025), and future studies should include multi-kingdom microbiota profiling.

#### 2.2.2 Insufficient exploration of SCFA-mediated signaling pathways

Liu et al. (2025) demonstrated that PWP increases intestinal SCFA levels and upregulates the SCFA receptor GPR43, but they did not investigate the downstream signaling pathways by which SCFAs exert their effects. SCFAs exert anti-inflammatory and barrier-protective effects through multiple mechanisms. In the GPR43/GPR41 signaling pathway, activation of G protein coupled receptors inhibits NF-κB mediated inflammation and enhances expression of tight junction proteins (Kreuter et al., 2019). By inhibiting histone deacetylase (HDAC), particularly butyrate can inhibit HDAC activity and promote the expression of anti-inflammatory genes and autophagy-related genes (Donohoe et al., 2011). Besides, SCFAs serve as energy substrates for colonocytes, maintaining mitochondrial function and barrier integrity (Hamed et al., 2023).

A recent study by Hamed et al. (2023) showed that butyrate protects intestinal epithelial barrier function by activating GPR43 and restoring mitochondrial morphology. Liu et al.’s study could have strengthened its mechanistic insights by investigating these pathways—for example, by using GPR43 knockout mice to determine whether PWP’s effects are dependent on GPR43. Furthermore, given the synergistic effect of SCFAs with TCM formulas observed by Guo et al. (2025), future studies could explore whether combining PWP with SCFAs (e.g., sodium propionate) enhances the activation of these signaling pathways, thereby improving therapeutic efficacy.

#### 2.2.3 Lack of long-term efficacy and safety evaluation

The study evaluated PWP’s efficacy over a short period and did not assess long-term outcomes such as relapse rates or potential side effects. In clinical practice, UC is a chronic disease requiring long-term management, and short-term animal studies may not reflect the durability of PWP’s effects. Additionally, while PWP is generally considered safe, high doses of its components may have hepatotoxic or neurotoxic effects (Luo et al., 2019). Future studies should include long-term administration and monitor serum biochemical markers to evaluate safety.

Furthermore, the study used a single dose of PWP without exploring dose-response relationships. Dose optimization is critical for clinical translation, as excessive doses may increase side effects while suboptimal doses may reduce efficacy. Drawing inspiration from Guo et al.’s (2025) finding that SCFAs reduce TCM formula dosage, future studies could investigate whether combining PWP with SCFAs allows for dose reduction, minimizing potential side effects while maintaining efficacy.

#### 2.2.4 Limited investigation of autophagy mechanisms

While Liu et al. showed that PWP promotes colonic autophagy, they did not clarify the specific type of autophagy involved or its upstream regulators. Autophagy in UC includes Xenophagy (Sharma et al., 2018), Mitophagy (Wang et al., 2024a). The PI3K/AKT/mTOR pathway inhibits autophagy by phosphorylating ULK1 (Kim et al., 2011), but Liu et al. did not measure ULK1 phosphorylation or mitophagy markers. Additionally, the study did not determine whether autophagy is required for PWP’s effects—for example, by using autophagy-deficient mice to test if PWP’s efficacy is abrogated.

#### 2.2.5 Lack of clinical translation data

Liu et al.’s study was conducted in mice, and the authors did not provide data on PWP’s efficacy in human UC patients. While animal models are valuable for mechanistic research, they have limitations: HFD-DSS-induced UC in mice does not fully recapitulate the chronic, relapsing nature of human UC, and interspecies differences in gut microbiota composition may affect PWP’s efficacy (Thomas et al., 2023). Future studies should include ex vivo experiments using human colonic organoids or fecal samples from UC patients to validate PWP’s effects on human gut microbiota and epithelial cells. Additionally, building on Guo et al.’s (2025) clinical translation-oriented approach, phase II clinical trials of PWP could include an arm testing PWP-SCFA combination therapy, evaluating both efficacy and safety in human patients.

To bridge this gap, future studies should first conduct ex vivo experiments using human colonic organoids (derived from UC patients or healthy controls) to validate PWP’s effects on barrier function (e.g., measuring transepithelial electrical resistance) and inflammation (e.g., quantifying pro-inflammatory cytokine secretion) (Li et al., 2021). Additionally, fecal samples from UC patients could be used in *in vitro* fermentation models to assess whether PWP modulates human gut microbiota composition and SCFA production—consistent with the approach used by Thomas et al. (2023) to link diet and UC therapeutic efficacy. Building on Guo et al.’s (2025) clinical translation-oriented approach, phase II clinical trials of PWP could include an arm testing PWP-SCFA combination therapy, with endpoints such as clinical remission rate (based on the Mayo Score), endoscopic improvement, and changes in fecal SCFA levels and microbiota composition (Haskey et al., 2023). These steps are critical to translate preclinical findings into clinical practice.

## 3 Discussion

Liu et al.’s (2025) study represents a significant advance in understanding the mechanism of PWP in HFD-induced colonic inflammation, providing a scientific basis for its clinical application in UC. However, to fully realize PWP’s potential, future research should address the limitations outlined above through the following strategies:

1. Employ multi-omics to decipher gut microbiota function

To clarify how PWP modulates SCFA production and inflammatory responses, a stepwise multi-omics integration strategy is recommended: (1) Metagenomic sequencing: Analyze changes in microbial functional genes to determine whether PWP’s effect on SCFAs is driven by functional gene regulation or taxonomic shifts. This can be complemented by PICRUSt2 (Phylogenetic Investigation of Communities by Reconstruction of Unobserved States) to predict functional pathways from 16S rDNA data, with metagenomic sequencing validating key predictions (Ma et al., 2024). (2) Metabolomics: Use liquid chromatography-mass spectrometry (LC/MS) to profile global intestinal metabolites, focusing on SCFAs and their downstream metabolites, as well as other inflammation-related metabolites. Integrate metabolomics data with metagenomic results to identify microbiota-metabolite co-expression networks (Jiang et al., 2025). (3) Transcriptomics: Perform RNA sequencing of colonic tissues to identify PWP-regulated genes involved in the PI3K/AKT/mTOR pathway and autophagy. Use weighted gene co-expression network analysis (WGCNA) to link transcriptional changes to microbiota and metabolite alterations, providing a comprehensive view of molecular changes (Zheng et al., 2022).

2. Investigate SCFA-mediated signaling pathways

Use GPR43 or GPR41 knockout mice to determine whether PWP’s effects on barrier function and inflammation require these receptors (Kreuter et al., 2019). Measure HDAC activity in colonic tissues and use HDAC inhibitors/activators to test if PWP’s autophagy-promoting effects are mediated by HDAC inhibition (Donohoe et al., 2011). Inspired by Guo et al.’s (2025) work, test the efficacy of PWP combined with sodium propionate or other SCFAs, evaluating whether this combination enhances GPR43 activation, HDAC inhibition, or mitochondrial function (Hamed et al., 2023).

3. Conduct Long-term efficacy and safety evaluation

Use a chronic DSS model to evaluate PWP’s ability to prevent relapse (Li et al., 2022). Monitor serum liver and kidney function markers, and perform histopathological analysis of major organs to evaluate long-term safety (Luo et al., 2019). Test multiple PWP doses alone and in combination with sodium propionate to identify the minimum effective dose and optimal therapeutic window (Guo et al., 2025).

4. Clarify autophagy mechanisms

Measure markers of xenophagy and mitophagy to determine the specific type of autophagy promoted by PWP (Sharma et al., 2018; Wang et al., 2024a). Use Atg5 or ULK1 knockout mice to test if PWP’s anti-inflammatory effects are abrogated in the absence of autophagy (Kim et al., 2011).

5. Advance clinical translation

Treat human colonic organoids with PWP or PWP-modulated fecal microbiota to validate its effects on barrier function and inflammation (Thomas et al., 2023). Conduct phase II randomized controlled trials (RCTs) to evaluate PWP’s efficacy in UC patients, with endpoints including clinical remission rate, endoscopic improvement, and changes in fecal SCFA levels and microbiota composition (Haskey et al., 2023; Wang et al., 2024b). Include an arm testing PWP + sodium propionate to evaluate dosage reduction and synergistic efficacy (Guo et al., 2025). Given the role of HFD in UC pathogenesis, test the efficacy of PWP combined with a low-fat, high-fiber diet (e.g., Mediterranean diet) in both animal models and clinical trials (Fritsch et al., 2021; Haskey et al., 2023).
